# Suitability of Yin Yang 1 transcript and protein levels for biomarker studies in B cell non-Hodgkin lymphoma

**DOI:** 10.1186/s40364-018-0126-y

**Published:** 2018-03-13

**Authors:** Jéssica Arribas Arranz, Dalia Nilufar Winter, Hans Günter Drexler, Sonja Eberth

**Affiliations:** Department of Human and Animal Cell Lines, Leibniz-Institute DSMZ, German Collection of Microorganisms and Cell Cultures, Inhoffenstrasse 7 B, 38124 Braunschweig, Germany

**Keywords:** Biomarker, B-NHL, DLBCL, Gene expression, Half-life, Prognostic factor, YY1

## Abstract

**Background:**

Yin Yang 1 (YY1) is a transcription factor that plays an important role during all stages of B cell differentiation. Several studies reported upregulation of YY1 in B cell derived lymphoma, indicating that it might act as an oncogene. Furthermore, aberrant YY1 expression has been associated with survival in some entities of B cell non-Hodgkin lymphoma (B-NHL), suggesting that YY1 could be a valuable biomarker in B-NHL. However, studies are controversial and methodologically disparate, partially because some studies are based on transcript levels while others rely on YY1 protein data. Therefore, we aimed to investigate the dependence of YY1 protein levels on *YY1* transcription.

**Methods:**

A panel of human cell lines representing different B-NHL subtypes was used to test for the correlation of *YY1* mRNA and protein levels which were determined by quantitative PCR and immunoblotting. To analyze *YY1* mRNA and YY1 protein stability cells were treated with actinomycin-D and cycloheximide, respectively. siRNAs were transfected to knockdown *YY1*. Kaplan-Meier survival analyses were performed with data from published patient cohorts. Pearson’s correlation analyses were assessed and statistical power was examined by Student’s t-test.

**Results:**

In the analyzed panel of B-NHL cell lines *YY1* transcript levels do not correlate with their cellular protein amounts. YY1 protein levels were unaffected by transient block of transcription or by targeting *YY1* mRNA using siRNA. Additionally, global inhibition of translation up to 48 h did not alter protein levels of YY1, indicating that YY1 is a highly stable protein in B-NHL. Furthermore, in a retrospective analysis of two different B-NHL cohorts, *YY1* transcript levels had no impact on patients’ survival probabilities.

**Conclusions:**

Our results point out the necessity to focus on YY1 protein expression to understand the potential role of YY1 as an oncogene and to unravel its suitability as clinical biomarker in B-NHL.

**Electronic supplementary material:**

The online version of this article (10.1186/s40364-018-0126-y) contains supplementary material, which is available to authorized users.

## Background

The transcription factor Yin Yang 1 (YY1) is an ubiquitously expressed zinc finger protein of the GLI-Kruppel class that fulfills multiple roles in development, proliferation, apoptosis and differentiation [1]. According to context, YY1 can act either as a transcriptional repressor or activator. YY1 works as a transcription factor which has DNA and RNA binding properties [2]. Across the entire genome, YY1 binds to both gene regulatory elements and RNA species transcribed from these elements which was postulated to act as positive-feedback for YY1 binding to these regions [3]. Its specific function depends on its localization and is determined by its network, thus its interaction with other proteins, DNA and RNA [4].

YY1 is known to be a critical regulator of early B cell development. Liu et al. revealed in mice that the deletion of *YY1* in B lymphocyte progenitors results in the arrest at the pro-B cell stage [5]. Moreover, YY1 emerged as a new key transcription factor of the germinal center (GC) reaction in secondary lymphoid tissues [6], the place where B cell maturation and expansion occurs. Several subtypes of B cell non-Hodgkin lymphoma (B-NHL) are derived from GC B cells, for example diffuse large B cell lymphoma (DLBCL), which is the most prevalent subtype. In fact, B-NHL is a heterogeneous group of lymphomas that are derived from B cells and B-NHL sub-entities mirror the normal stage of physiological B cell differentiation [7].

YY1 is deregulated and implicated in several cancers including solid tumors and hematological malignancies like B-NHL [8–10]. In accordance to these observations, YY1 is discussed as a biomarker and potential drug target in several tumor types [11–14].

Compared with reactive lymph nodes, samples from DLBCL and follicular lymphoma (FL), another B-NHL subtype, have significantly higher *YY1* transcript levels and an increased *YY1* mRNA level has been associated with shorter survival of the patients [15]. Castellano et al. reported about enhanced *YY1* transcript levels in primary high-grade lymphoma samples from Burkitt lymphoma (BL) and DLBCL in comparison to primary low-grade B cell malignancies and normal B cells [16]. Furthermore, overexpression of YY1 was detected in several B-NHL cell lines and in silico mRNA data analyses revealed that the altered expression of *YY1* might promote B cell transformation and contribute to tumor progression [16]. Moreover, Vega et al. reported that increased expression of YY1 might contribute to chemoresistance in B-NHL cell lines [17]. In contrast, a study based on YY1 protein status in primary B-NHL samples showed that higher YY1 protein levels could be used as a clinical prognostic factor for favorable outcome in FL patients [18].

Altogether, current knowledge suggests that YY1 regulates various B cell stage-specific functions and that it might act as an oncogene if deregulated in B cells. Additionally, YY1 might serve as a valuable prognostic biomarker in some B-NHL subtypes. However, our knowledge about the consequences of YY1 deregulation in B-NHL is yet incomplete and data about the correlation of YY1 expression levels with clinical outcome are controversial.

The aim of this study is to elucidate the controversies published about YY1, investigating the relationship between *YY1* transcript levels and protein abundance in B-NHL cell lines.

## Methods

### Cell culture and inhibitor treatment

Cell lines were taken from the authenticated stocks of the DSMZ cell line bank (Leibniz- Institute DSMZ – German Collection of Microorganisms and Cell Cultures, Braunschweig, Germany). One normal lymphoblastoid cell line (NC-NC) was taken as a non-malignant control and 13 B-NHL cell lines were selected based on their original classification: Burkitt lymphoma (BL: RAMOS and BL-2), activated B cell-like diffuse large B cell lymphoma (ABC DLBCL: OCI-LY3, U2932-R1 and U2932-R2), germinal center B cell-like diffuse large B cell lymphoma (GCB DLBCL: SU-DHL-10, NU-DUL-1, OCI-LY7 and WSU-FSCCL), follicular lymphoma (FL: SC-1), or as mantle cell lymphoma (MCL: REC-1, GRANTA-519 and JEKO-1) cell lines. Detailed references and cultivation protocols have been described previously [19, 20].

Actinomycin D (ACT-D, Sigma-Aldrich) was used as a general transcriptional inhibitor. Cells were seeded at a density of 1x10^6^cells/mL, ACT-D (previously diluted in water) was added at a final concentration of 5 μg/mL. At 0, 0.5, 1, 2, 4 and 6 h of treatment; or at 0, 6, 10, 24, 34 and 48 h of treatment; cells were harvested for RNA or protein. Cycloheximide (CHX, Sigma-Aldrich) was used to block translation in the cells. Cells were seeded at a density of 1x10^6^cells/mL, CHX (pre-diluted in water) was added at a final concentration of 100 μg/mL. At 0, 6, 10, 24, 34 and 48 h of treatment cells were harvested for protein.

### Transient *YY1* knockdown

Small-interfering RNA (siRNA) against the target gene *YY1* (siYY1_3: SI03650318 and siYY1_5: SI03027304; Qiagen) or scrambled control (siSCR: SI03650318; Qiagen) were transfected using Lonza Nucleofection Kit (SF Cell Line 4D-Nucleofector® X Kit L; Lonza) and the 4D Nucleofector device from Lonza (100 pmol siRNA/5 × 10^6^ cells) according to the manufacturer’s instructions (programs: DG-137 for U2932-R2, and CM-138 for RAMOS and SC-1, chosen based on the cell viability and reached efficiency determined previously by transfection of a GFP plasmid). After the nucleofection process, cells were incubated in pre-warmed culture medium for 24 and 48 h at 37 °C in 5% CO_2_, before they were harvested to prepare RNA or protein.

### Ectopic overexpression of YY1

YY1 expression vector (YY1.V) encoding the full-length cDNA of human *YY1* (SC118004; Origene) or its corresponding empty vector pCMV6 (EV) were transfected into U2932-R1 cells using Lonza Nucleofection Kit (SF Cell Line 4D-Nucleofector® X Kit L; Lonza) and the 4D Nucleofector device from Lonza (5 μg plasmid/5 × 10^6^ cells) according to the manufacturer’s instructions (program DG-137). After the nucleofection process, cells were incubated in pre-warmed culture medium for 24 and 48 h at 37 °C in 5% CO_2_, before they were harvested to prepare protein lysates.

### RNA isolation, cDNA synthesis and quantitative real-time PCR (qRT-PCR)

Preparation and isolation of total RNA from cell samples was performed using QIAzol Lysis reagent (Qiagen) and miRNeasy Mini kit (Qiagen) following the manufacturer’s instructions. One microgram of total RNA was reverse transcribed to cDNA using the Superscript II First-strand Synthesis Kit (Invitrogen). Gene expression of *YY1, MYC*, *GAPDH* and *RPS9* was analyzed by qRT-PCR in a reaction volume of 20 μL using 2 ng of template and SsoFast™ EvaGreen® Supermix (Biorad), according to the manufacturer’s protocol in the Applied Biosytems 7500 Fast Real-Time PCR System using the thermal profile: 2 min 95 °C followed by 40 cycles of 2 s 95 °C, 25 s 60 °C. The specificity of reaction was verified by subsequent melt curve analysis to exclude the presence of by-products or primer-dimer formation. Used primer pairs are specified in Table 1. All primer pairs were pre-selected based on their performance in a standard curve determined on a series of consecutive dilutions. Each qRT-PCR reaction was performed in triplicate. The relative expression levels of the target genes were normalized using the reference genes *GAPDH* and *RPS9*, or only *GAPDH* (when comparing samples derived from one cell line) after checking that C_T_ values are in linear dynamic range, and calculated with the ∆∆C_T_ method.

**Table 1:** Primers for qRT-PCR

Gene		Primer sequence (5´➔3´)	Amplification efficiency/r^2^
*YY1*	fwd	ACCTGGCATTGACCTCTCAG	101% / 0.996
	rev	TGCAGCCTTTATGAGGGCAAG	
*GAPDH*	fwd	TGGGTGTGAACCATGAGAAG	101% / 0.999
	rev	TCCACGATACCAAAGTTGTCA	
*RPS9*	fwd	GGGAAGCGGAGCCAACATG	98% / 0.998
	rev	GTTTGTTCCGGAGCCCATACT	

### Protein lysates, quantification and western blot analysis

Total cell lysates were prepared in RIPA buffer containing protease and phosphatase inhibitors (Roche). Fifteen μg of protein were separated on 10–15% polyacrylamide SDS gels and electrotransferred onto PVDF membranes (Biorad) using wet tank sandwich method. Membranes were blocked and probed with the respective primary antibody: anti-YY1 (1:5000, rabbit monoclonal IgG EPR4652: ab109237; Abcam), anti-YY1 (1:5000, mouse monoclonal IgG_1_ H10: sc7341; Santa Cruz Biotechnology), anti-YY1 (1:5000, rabbit polyclonal IgG H414: sc1703; Santa Cruz Biotechnology), anti-MYC (1:10000, rabbit monoclonal IgG Y69: ab32072; Abcam), anti-GAPDH (1:10000, mouse monoclonal IgG_1_ 6C5: ab8245; Abcam), anti-histone H3 (1:1000, rabbit polyclonal IgG: #9715; Cell Signaling). Secondary antibodies were purchased from GE Healthcare Life Sciences and used at 1:10000 (anti-mouse IgG HRP-linked NXA931, anti-rabbit IgG HRP-linked NA934). Protein bands were detected by chemiluminescence (Western lightning Plus ECL Solution; Perkin Elmer) in the Advanced Fluorescence Imager machine (Intas) and protein signal quantification was performed by densitometry analysis using ImageJ software. Pearson’s correlation coefficient was determined by Microsoft Excel software (Microsoft Office Professional Plus 2010).

### Survival analyses

Data sets from DLBCL patients from the Molecular Mechanism in Malignant Lymphoma (MMML) cohort (*n* = 399) [21], as well as from cyclophosphamide, doxorubicin, oncovin and prednisone (CHOP) chemotherapy treatment (*n* = 181) and CHOP in combination with rituximab (R-CHOP) (*n* = 233) treated DLBCL cohorts [22] were taken for Kaplan-Meier survival analyses using the web portal LYMMML (version 1.11.13) of the Institute of Functional Genomics at the University of Regensburg (http://lymmml.ur.de). Samples were split according to their *YY1* mRNA expression levels in one group above and one group below the median *YY1* value.

## Results

### *YY1* transcript and protein levels do not correlate in B-NHL cell lines

**Fig. 1:**
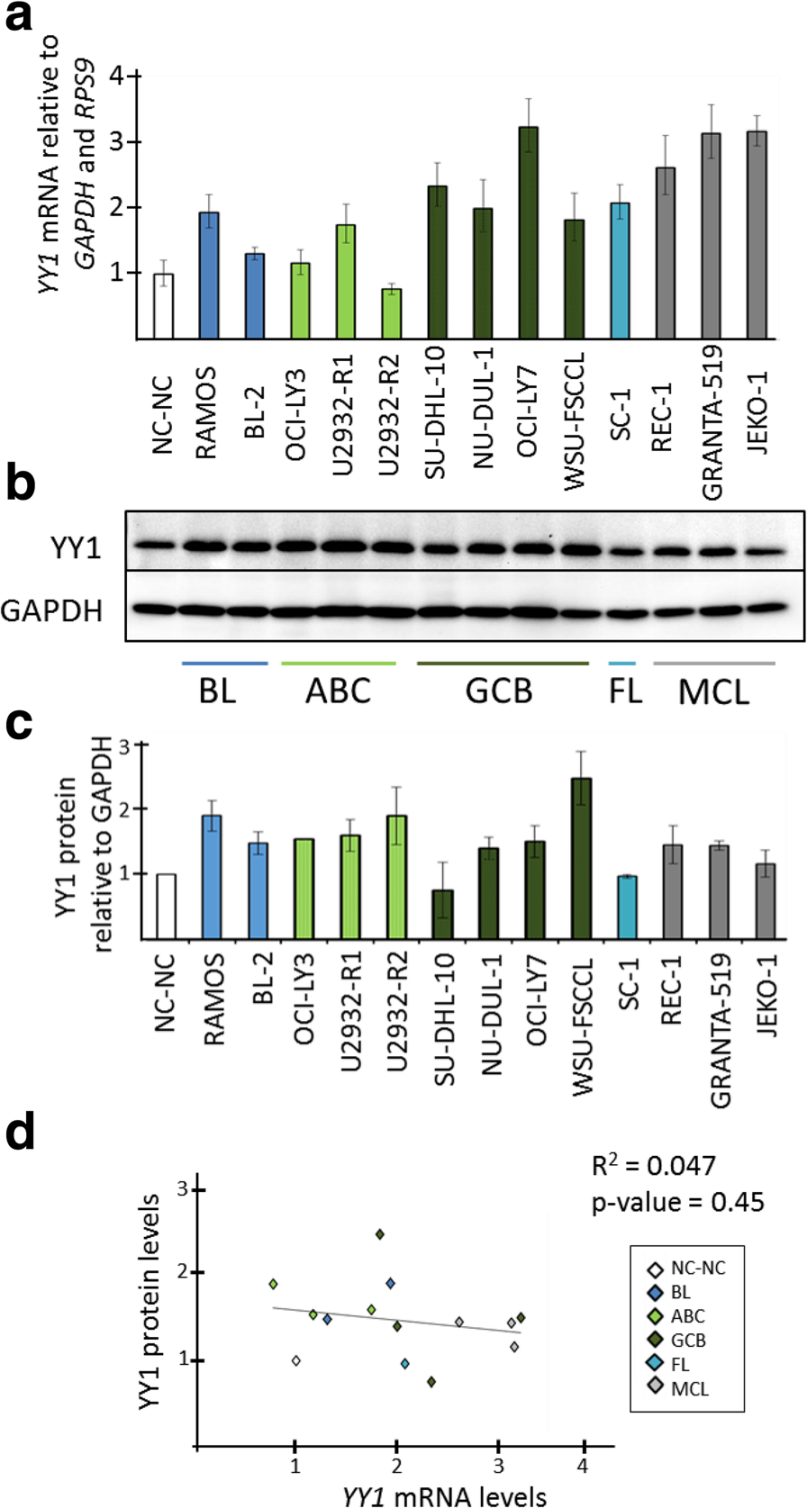
YY1 expression in B-NHL cell lines and in a B lymphoblastoid cell line (NC-NC) as non-cancer control. **a** *YY1* mRNA quantification relative to *GAPDH* and *RPS9* by qRT-PCR from two biological replicates. NC-NC was set to 1. Error bars indicate 95% confidence interval of the mean expression. **b** Representative western blot analysis of YY1 protein in total lysates. GAPDH was used as loading control. **c** YY1 protein signal quantification in total lysates relative to GAPDH of two independent western blot analyses. NC-NC was set to 1. **d** Relative levels of *YY1* mRNA plotted against relative YY1 protein amounts. Correlation coefficient is represented as R^2^ value. Different colors refer to the different lymphoma subtypes. BL: Burkitt lymphoma; ABC: activated B cell-like DLBCL; GCB: germinal centre B cell-like DLBCL; FL: follicular lymphoma; MCL: mantle cell lymphoma

*YY1* expression was evaluated at mRNA and protein level in a panel of 13 human B-NHL cell lines representing different subtypes (BL, DLBCL, FL and mantle cell lymphoma, MCL) and a normal B lymphoblastoid cell line (NC-NC). Quantitative real-time PCR (qRT-PCR) analysis showed that the amount of *YY1* was higher in the malignant B-NHL cell lines analyzed compared with the normal cell line NC-NC (Fig. 1a). The strongest *YY1* expression was detected in the DLBCL cell line OCI-LY7 with a 3-fold increase in comparison to NC-NC. However, no subtype-specific expression pattern was apparent at mRNA level within this panel of cell lines. Before analyzing the YY1 protein amount in this set of cell lines, three different commercial anti-YY1 antibodies (two monoclonal: ab109237 and sc7341; and one polyclonal: sc1703) were tested for their specificity. All three antibodies performed uniformly and were able to detect the increase of a protein of the reported size of YY1 after ectopic overexpression of YY1 in cell line U2932-R1, thus proving their specificity in YY1 detection (Additional file 1: Figure S1). All following experiments were performed with the monoclonal anti-YY1 antibody ab109237. Subsequent western blot analyses of lysates from the panel of B-NHL cell lines revealed the presence of YY1 in all of the examined samples, with varying YY1 signal intensity among the different cell lines (Fig. 1b, c). The highest YY1 level was detected in the DLBCL cell line WSU-FSCCL. According to densitometric evaluation YY1 amounts differed 2.5-fold in maximum between the tested cell lines (Fig. 1c). Thus, the range of *YY1* expression was smaller at protein level than at transcript level. Remarkably, there was not a direct correlation between *YY1* mRNA and protein abundance. For example, transcript level of *YY1* in cell line U2932-R2 was comparable to the *YY1* level in B lymphoblastoid cell line NC-NC, but the amount of YY1 protein was clearly higher in U2932-R2 than in NC-NC. And the other way around, cell line SU-DHL-10 had a high *YY1* mRNA level among the cell lines analyzed but this was not visible at protein level, in which SU-DHL-10 was similar to NC-NC. This lack of correlation is demonstrated in Fig. 1d in which relative levels of *YY1* mRNA are plotted against relative YY1 protein amounts with a coefficient of determination (R^2^) of 0.047. Therefore, these results indicate that there is not a direct correlation between *YY1* mRNA and protein levels in B-NHL.

### Global inhibition of transcription and translation has no influence on YY1 protein levels

**Fig. 2:**
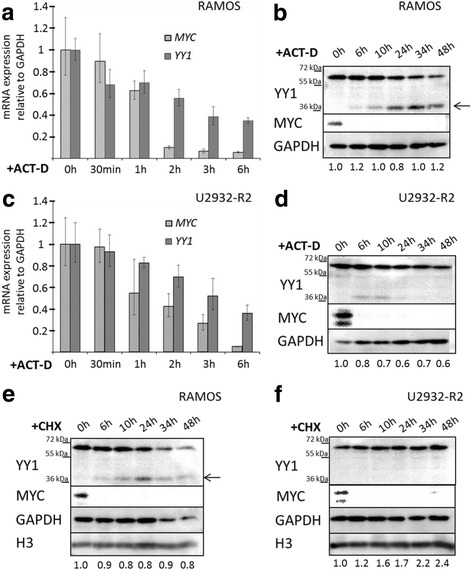
Cellular stability of *YY1* mRNA and protein. Influence of the transcriptional inhibitor actinomycin-D (ACT-D, 5 μg/mL) (**a**-**d**) and of the translational inhibitor cycloheximide (CHX, 100 μg/mL) (**e**, **f**) on YY1 expression in the B-NHL cell lines RAMOS (**a**, **b** and **e**) and U2932-R2 (**c**, **d** and **f**). **a** *YY1* mRNA quantification relative to *GAPDH* by qRT-PCR after 0 h, 30 min, 1 h, 2 h, 3 h and 6 h of ACT-D treatment of RAMOS and (**c**) of U2932-R2. Error bars indicate 95% confidence interval of the mean expression. *MYC* levels were analyzed as a positive control for a fast turnover mRNA. **b** Western blot analysis of YY1 protein after 0 h, 6 h, 10 h, 24 h, 34 h and 48 h of ACT-D treatment in RAMOS and (**d**) in U2932-R2. MYC was used as a positive control for a fast turnover protein and GAPDH as loading control. The arrow indicates the caspase-cleaved YY1 form. **e** Western blot analysis of YY1 protein after 0 h, 6 h, 10 h, 24 h, 34 h and 48 h CHX treatment of RAMOS and (**f**) of U2932-R2. MYC expression was determined as a positive control for a fast turnover protein and GAPDH and histone 3 (H3) served as loading controls. The arrow indicates the caspase-cleaved YY1 form. Numbers underneath the blots refer to the relative amount of YY1 normalized to GAPDH levels according to densitometric analyses of the blots. The 0 h sample was set to 1 at each time point, treatment and cell line

In order to analyze cellular stability of *YY1* mRNA and protein, two B-NHL cell lines representing different subtypes, namely BL cell line RAMOS and DLBCL cell line U2932-R2, were selected and maintained in presence of the transcriptional inhibitor actinomycin-D (ACT-D) for 30 min up to 48 h. Subsequently, RNA and total protein were isolated and subjected to qRT-PCR and western blot analyses, respectively (Fig. 2a-d). Because MYC has a fast turnover rate [23], stability of *MYC* mRNA and protein was determined in parallel to control the activity of the inhibitor. As expected, ACT-D treatment resulted in a decrease of *YY1* mRNA levels in RAMOS (Fig. 2a) and U2932-R2 (Fig. 2c) in the analyzed time frame of 6 h in which the C_T_ values of the endogenous control *GAPDH* remained stable. The stability of *GAPDH* is in accordance with previous data [24]. Estimated *YY1* mRNA half-lives are in the same range in both B-NHL cell lines studied (~ 3,5 h for RAMOS and ~ 3 h for U2932-R2). However, inhibition of transcription through long term treatment (48 h) with ACT-D did not lead to a decrease of YY1 protein in RAMOS and U2932-R2 (Fig. 2b, d), suggesting high stability of YY1 protein in the B-NHL cell lines. Indeed, the stability of YY1 was comparable to that of the endogenous control GAPDH. Note that the decrease of full-length YY1 in RAMOS is due to cleavage of YY1 (arrow in Fig. 2b) by caspases as a result of ACT-D triggered apoptosis, confirming a phenomenon which was reported before [25]. In order to examine the turnover rate of YY1 protein, we inhibited global protein translation in B-NHL cell lines using cycloheximide (CHX) (Fig. 2e-f). No significant decrease of YY1 protein was observed in RAMOS and U2932-R2 within the first 24 h of treatment. In U2932-R2 YY1 levels remained stable even after 48 h (Fig. 2f). Nevertheless, as shown before a reduction of YY1 full-length protein related to activation of apoptosis was detected in RAMOS (Fig. 2e) which appeared much more sensitive towards the inhibitors used than U2932-R2. Consequently, YY1 protein levels seem to be independent of de novo protein synthesis for up to 48 h and are thus highly stable in B-NHL cell lines.

### Transient knockdown of *YY1* has a marginal effect on YY1 protein levels

**Fig. 3:**
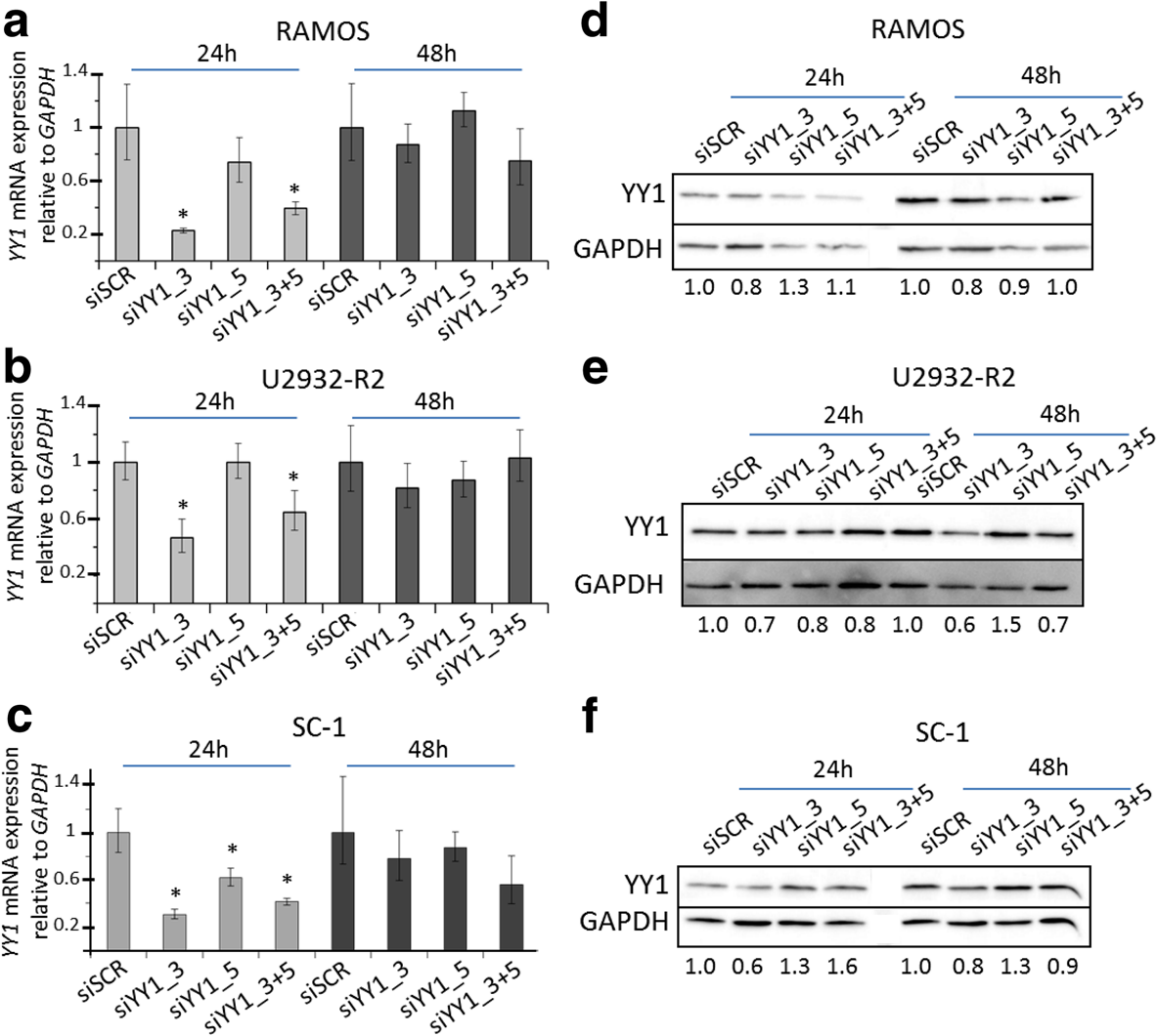
Transient knockdown of *YY1* by siRNA in B-NHL. Cell lines RAMOS (**a**, **d**), U2932-R2 (**b**, **e**) and SC-1 (**c**, **f**) were transfected by nucleofection with two different siRNAs targeting *YY1* (siYY1_3, siYY1_5) and the combination of both (siYY1_3 + 5), as well as a scrambled siRNA (siSCR) control. **a** *YY1* mRNA quantification relative to *GAPDH* by qRT-PCR of the 24 h and 48 h post nucleofection samples in RAMOS, **b** in U2932-R2 and **c** in SC-1. The scrambled control was set to 1 at each time point. Error bars indicate 95% confidence interval of the mean expression. Asterisks indicate a *p*-value < 0.05 in case of a significant difference between the mean of a siRNA treated sample compared to the mean of the scrambled control according to t-test. **d** Western blot analysis of YY1 protein of the same samples shown in (**a**). **e** Western blot of YY1 protein of the same samples shown in (**b**). **f** Western blot of YY1 protein of the same samples shown in (**c**). GAPDH was used as loading control. Numbers refer to the relative amount of YY1 normalized to GAPDH levels according to densitometric analyses of the blots. The scrambled control was set to 1 at each time point

To avoid unspecific effects like apoptosis of global inhibitors, we next assessed the influence of *YY1* knockdown on YY1 protein levels using small interference RNA (siRNA) against *YY1* mRNA in cell lines RAMOS, U2932-R2 and SC-1. Twenty-four and 48 h after nucleofection, RNA and protein levels of YY1 were analyzed (Fig. 3). Although a significant knockdown of *YY1* at mRNA level, mainly by siRNA siYY1_3, was detected 24 h post transfection in all three cell lines (Fig. 3a-c), these alterations in *YY1* mRNA abundance only had marginal influence on YY1 protein levels (Fig. 3d-f). Thus, in summary, the small effect of *YY1* knockdown on YY1 protein levels further demonstrates the high stability of YY1 protein in B-NHL cell lines and is in line with the results obtained after global inhibition of transcription.

### Survival probability of DLBCL patients is independent from *YY1* mRNA level

**Fig. 4:**
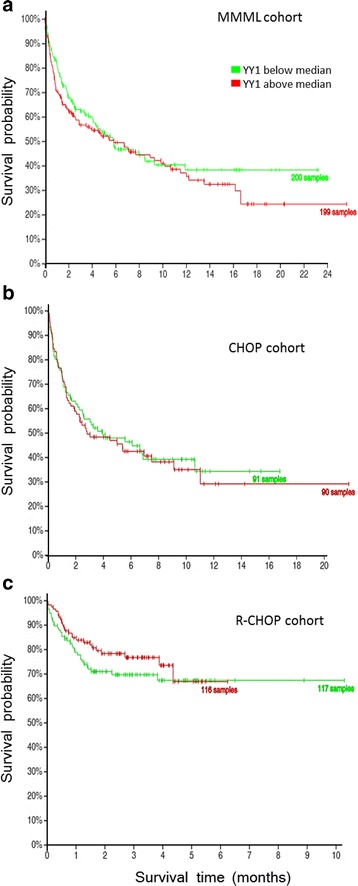
Survival probabilities of DLBCL patients in respect to their *YY1* mRNA expression levels. Samples with *YY1* levels below median are depicted in green, samples expressing *YY1* above median level are painted in red. Analyses from the MMML cohort (**a**) from Hummel et al. [21] and from the CHOP treated (**b**) and R-CHOP treated (**c**) DLBCL cohorts from Lenz et al. [22]. Survival analyses were performed with, and taken from http://lymmml.ur.de

Finally, correlation of *YY1* mRNA expression with patient survival was evaluated using transcriptomic and clinical data from two different DLBCL patient cohorts that are publicly available [21, 22]. Kaplan-Meier survival analyses were performed after splitting the cohorts according to their *YY1* mRNA expression levels in one group above and one group below the median *YY1* value (Fig. 4). In the analyzed cohorts of DLBCL patients, the expression level of *YY1* mRNA has no impact on survival of the patients. This observation is also independent of the treatment regimen the patients received, as we compared patients with high and low *YY1* mRNA levels treated with CHOP alone (Fig. 4b) and CHOP combined with rituximab (R-CHOP) (Fig. 4c).

## Discussion

YY1 is an important transcription factor during all stages of B cell differentiation [26]. Several studies reported upregulation of YY1 in B cell derived lymphoma, including BL and DLBCL [15, 16], indicating that it might act as an oncogene in B-NHL. Consequentially, YY1 was tested as a prognostic biomarker in subtypes of B-NHL and has been discussed as a putative therapeutic target (reviewed in [27]).

Using a panel of different B-NHL cell lines representing several subtypes we demonstrate in this study that *YY1* transcript levels do not correlate with YY1 protein amount in these cells. Importantly, neither transient inhibition of transcription nor block of translation resulted in a significant decrease of YY1 protein levels, rendering YY1 a highly stable protein in B-NHL. Thus, our results indicate that cellular abundance of YY1 protein is primarily modulated by post-translational mechanisms that are involved in regulating YY1 protein stability and degradation.

Interestingly, the ubiquitin ligase Smurf2, that was shown to regulate protein stability of YY1 in DLBCL, was reported to be downregulated in primary DLBCL samples which in addition correlated with inferior survival in these patients [28]. Nevertheless, there is the need to further study factors affecting YY1 degradation in B-NHL.

The mRNA levels of a gene do not always correlate with its cellular protein amounts (reviewed in [29]). There are some examples for YY1 from non-hematological cell types. Walowitz et al. analyzed gene expression and protein level of YY1 during the differentiation of rat skeletal and cardiac muscle cells and found that although *YY1* mRNA levels did not change significantly, the YY1 protein levels decreased sharply [30]. They found out that YY1 is a substrate for Calpain II and also for ubiquitination leading to degradation via the 26S proteasome pathway [30]. Baritaki et al. observed the same expression pattern of YY1 protein among different groups of cervix pathologies, including cancer. However, they showed *YY1* mRNA overexpression only in one group compared to the others. Finally, they concluded that the overexpression of the protein YY1 may promote the progression of benign cervical lesions to malignant conditions [31]. In spite of these studies, positive correlation between *YY1* mRNA and protein levels has also been previously reported in other cancers. Matsumura et al. showed an elevated YY1 expression of both transcript and protein which positively correlated with enhanced survival of patients with ovarian cancer [32]. Zhang et al. provided more controversial results according to the outcome of pancreatic ductal adenocarcinoma (PDAC) patients. They detected YY1 overexpression in PDAC tissues compared to normal counterparts but, at the same time, the PDAC patients with higher levels of YY1 had a better outcome than the ones with lower levels [13].

Based on these observations and our data obtained from B-NHL cell lines, study results of YY1 expression in B-NHL patient samples will presumably, at least in part, depend on the methodological approach used. This became already apparent in FL in which it was reported that increased *YY1* mRNA levels are associated with a shorter survival in FL patients [15]. In contrast higher YY1 protein levels in FL patients could be used as a prognostic factor for favorable outcome [18]. In comparison to an immunological staining, transcriptomic data are nowadays often readily available from patient cohorts and can be used to study the correlation to clinical parameters. The herein analyzed cohorts of DLBCL patients showed no correlation between the expression level of *YY1* mRNA and survival probabilities, this observation was also independent from treatment regimens. However, other studies based on *YY1* transcript levels suggested that YY1 might play a role in DLBCL progression [15, 16]. At this moment, the reasons for the controversies observed for YY1 as a predictor of clinical outcome in hematological malignancies and among other cancers are unknown. Eventually, YY1 regulates specific tissue genes that are important for the response to therapy in each type of cancer; but we cannot ignore the fact of the high stability of YY1 protein and the relative independence of the protein levels from transcript levels in B-NHL cell lines. However, hitherto no parallel data of *YY1* transcript and protein levels are available from primary B-NHL samples. This demonstrates that indeed more data about YY1 protein status are necessary to pinpoint the potential oncogenic role of YY1 and its suitability as clinical biomarker in B-NHL.

## Conclusions

In summary, our results demonstrate that YY1 is a highly stable protein in B-NHL and that alterations in YY1 transcription and translation do not seem to have influence on YY1 protein levels at short term (up to 48 h). This points out the relevance to systematically include analyses of YY1 protein levels to prevent over-interpretation of alterations in *YY1* transcript levels. Future studies might include immunohistochemical stainings of YY1 to elucidate the potential role of YY1 protein as a prognostic biomarker and therapeutic target in B-NHL.

## Additional files

Additional file 1: **Figure S1.** Confirmation of anti-YY1 antibody specificity. Detection of YY1 with three different commercial antibodies directed against YY1 by western blot in lysates from U2932-R1 cells nucleofected with YY1 expression vector (YY1.V) or its corresponding empty vector (EV) harvested 24 and 48 h after transfection. The study-based anti-YY1 antibody (ab109237) and two other antibodies (sc7341 and sc1703) were tested. GAPDH served as loading control. (PDF 225 kb)

## Abbreviations

ABC DLBCL: Activated B cell-like diffuse large B cell lymphoma
ACT-D: Actinomycin-D
BL: Burkitt lymphoma
B-NHL: B cell non-Hodgkin lymphoma
CHOP: Cyclophosphamide, doxorubicin, oncovin and prednisone chemotherapy treatment
CHX: Cycloheximide
DLBCL: Diffuse large B cell lymphoma
FL: Follicular lymphoma
GC: Germinal center
GCB DLBCL: Germinal center B cell-like diffuse large B cell lymphoma
MCL: Mantle cell lymphoma
MMML: Molecular mechanism in malignant lymphoma
PDAC: Pancreatic ductal adenocarcinoma
R-CHOP: Cyclophosphamide, doxorubicin, oncovin and prednisone chemotherapy treatment combined with rituximab
siRNA: Small-interfering RNA
YY1: Yin yang 1

## References

[1] Deng Z, Cao P, Wan M, Sui G (2010). Yin Yang 1. A multifaceted protein beyond a transcription factor. Transcription.

[2] Jeon Y, Lee JT (2011). YY1 tethers Xist RNA to the inactive X nucleation center. Cell.

[3] Sigova AA, Abraham BJ, Ji X, Molinie B, Hannett NM, Guo YE (2015). Transcription factor trapping by RNA in gene regulatory elements. Science.

[4] Palko L, Bass HW, Beyrouthy MJ, Hurt MM (2004). The yin Yang-1 (YY1) protein undergoes a DNA-replication-associated switch in localization from the cytoplasm to the nucleus at the onset of S phase. J Cell Sci.

[5] Liu H, Schmidt-Supprian M, Shi Y, Hobeika E, Barteneva N, Jumaa H (2007). Yin Yang 1 is a critical regulator of B-cell development. Genes Dev.

[6] Green MR, Monti S, Dalla-Favera R, Pasqualucci L, Walsh NC, Schmidt-Supprian M (2011). Signatures of murine B-cell development implicate Yy1 as a regulator of the germinal center-specific program. Proc Natl Acad Sci U S A.

[7] Klein U, Dalla-Favera R (2008). Germinal centres: role in B-cell physiology and malignancy. Nat Rev Immunol.

[8] Zhang Q, Stovall DB, Inoue K, Sui G (2012). The oncogenic role of yin Yang 1. Crit Rev Oncog.

[9] Zaravinos A, Spandidos DA (2010). Yin yang 1 expression in human tumors. Cell Cycle.

[10] Arribas J, Castellví J, Marcos R, Zafón C, Velázquez A (2015). Expression of YY1 in differentiated thyroid cancer. Endocr Pathol.

[11] Shi J, Hao A, Zhang Q, Sui G (2015). The role of YY1 in oncogenesis and its potential as a drug target in cancer therapies. Curr Cancer Drug Targets.

[12] Bonavida B, Kaufhold S (2015). Prognostic significance of YY1 protein expression and mRNA levels by bioinformatics analysis in human cancers: a therapeutic target. Pharmacol Ther.

[13] Zhang J-J, Zhu Y, Xie K-L, Peng Y-P, Tao J-Q, Tang J (2014). Yin Yang-1 suppresses invasion and metastasis of pancreatic ductal adenocarcinoma by downregulating MMP10 in a MUC4/ErbB2/p38/ MEF2C-dependent mechanism. Mol Biol Cell.

[14] Wu L, Dong S, Ma X, Wang Z, Han B, Zou H (2017). YY1 promotes HDAC1 expression and decreases sensitivity of hepatocellular carcinoma cells to HDAC inhibitor. Oncotarget.

[15] Sakhinia E, Glennie C, Hoyland JA, Menasce LP, Brady G, Miller C (2007). Clinical quantitation of diagnostic and predictive gene expression levels in follicular and diffuse large B-cell lymphoma by RT-PCR gene expression profiling. Blood.

[16] Castellano G, Torrisi E, Ligresti G, Nicoletti F, Malaponte G, Travali S (2010). Yin Yang 1 overexpression in diffuse large B-cell lymphoma is associated with B-cell transformation and tumor progression. Cell Cycle.

[17] Vega MI, Jazirehi AR, Huerta-yepez S, Bonavida B (2005). Rituximab-induced inhibition of YY1 and Bcl-xL expression in Ramos non-Hodgkin’s lymphoma cell line via inhibition of NF-kB activity: role of YY1 and Bcl-xL in Fas resistance and chemoresistance, respectively. J Immunol.

[18] Naidoo K, Clay V, Hoyland JA, Swindell R, Linton K, Illidge T (2011). YY1 expression predicts favourable outcome in follicular lymphoma. J Clin Pathol.

[19] Drexler HG. Guide to leukemia-lymphoma cell lines. 2nd edition. Braunschweig; 2010.

[20] Quentmeier H, Amini RM, Berglund M, Dirks WG, Ehrentraut S, Geffers R (2013). U-2932: two clones in one cell line, a tool for the study of clonal evolution. Leukemia.

[21] Hummel M, Bentink S, Berger H, Klapper W, Wessendorf S, Barth TFE (2006). A biologic definition of Burkitt’s lymphoma from transcriptional and genomic profiling. N Engl J Med.

[22] Lenz G, Wright G, Dave S, Xiao W, Powell J, Zhao H (2008). Stromal gene signatures in large-B-cell lymphomas. N Engl J Med.

[23] Dani C, Blanchard JM, Piechaczyk M, El Sabouty S, Marty L, Jeanteur P (1984). Extreme instability of myc mRNA in normal and transformed human cells. Proc Natl Acad Sci U S A.

[24] Zhou H, Mazan-Mamczarz K, Martindale JL, Barker A, Liu Z, Gorospe M (2010). Post-transcriptional regulation of androgen receptor mRNA by an ErbB3 binding protein 1 in prostate cancer. Nucleic Acids Res.

[25] Krippner-Heidenreich A, Walsemann G, Beyrouthy MJ, Speckgens S, Kraft R, Thole H (2005). Caspase-dependent regulation and subcellular redistribution of the transcriptional modulator YY1 during apoptosis. Mol Cell Biol.

[26] Kleiman E, Jia H, Loguercio S, Su AI, Feeney AJ (2016). YY1 plays an essential role at all stages of B-cell differentiation. Proc Natl Acad Sci U S A.

[27] Bonavida B, Huerta-Yepez S, Baritaki S, Vega M, Liu H, Chen H (2011). Overexpression of yin Yang 1 in the pathogenesis of human hematopoietic malignancies. Crit Rev Oncog.

[28] Ramkumar C, Cui H, Kong Y, Jones SN, Gerstein RM, Zhang H (2013). Smurf2 suppresses B-cell proliferation and lymphomagenesis by mediating ubiquitination and degradation of YY1. Nat Commun.

[29] Liu Y, Beyer A, Aebersold R (2016). On the dependency of cellular protein levels on mRNA abundance. Cell.

[30] Walowitz JL, Bradley ME, Chen S, Lee T (1998). Proteolytic regulation of the zinc finger transcription factor YY1, a repressor of muscle-restricted gene expression. J Biol Chem.

[31] Baritaki S, Sifakis S, Huerta-Yepez S, Neonakis IK, Soufla G, Bonavida B (2007). Overexpression of VEGF and TGF-ß1 mRNA in pap smears correlates with progression of cervical intraepithelial neoplasia to cancer: implication of YY1 in cervical tumorigenesis and HPV infection. Int J Oncol.

[32] Matsumura N, Huang Z, Baba T, Lee PS, Barnett JC, Mori S (2009). Yin yang 1 modulates taxane response in epithelial ovarian cancer. Mol Cancer Res.

