# Supplementary material for: Suitability of Yin Yang 1 transcript and protein levels for biomarker studies in B cell non-Hodgkin lymphoma
Source: Biomark Res. 2018 Mar 13;6:11. doi: 10.1186/s40364-018-0126-y (PMC5850914; doi:10.1186/s40364-018-0126-y)
Supplement: Supplementary file 1 — Figure S1. Confirmation of anti-YY1 antibody specificity. Detection of YY1 with three different commercial antibodies directed against YY1 by western blot in lysates from U2932-R1 cells nucleofected with YY1 expression vector (YY1.V) or its corresponding empty vector (EV) harvested 24 and 48 h after transfection. The study-based anti-YY1 antibody (ab109237) and two other antibodies (sc7341 and sc1703) were tested. GAPDH served as loading control. (PDF 225 kb) [file 40364_2018_126_MOESM1_ESM.pdf]

Additional file 1

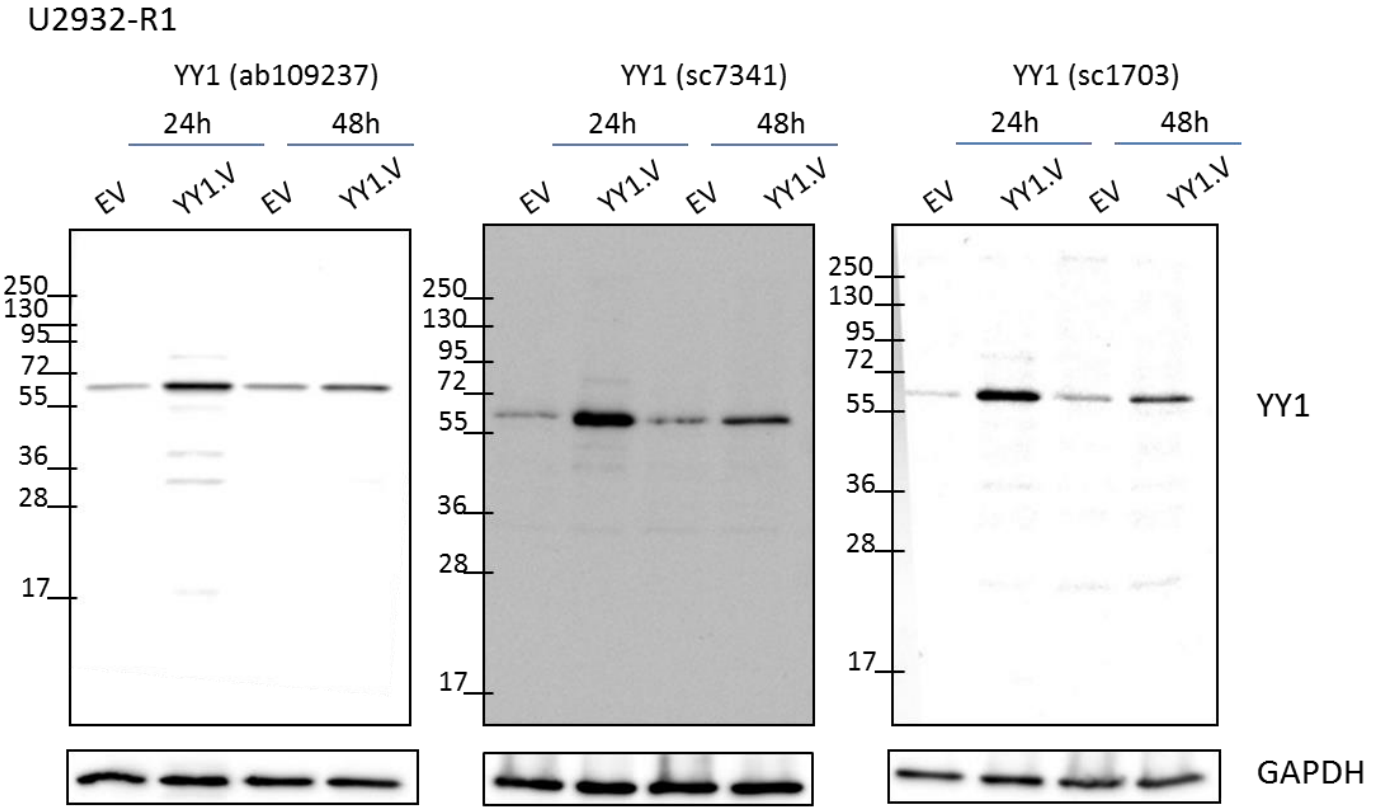

Figure S1
Confirmation of anti-YY1 antibody specificity. Detection of YY1 with three different commercial antibodies directed against YY1 by western blot in lysates from U2932-R1 cells nucleofected with YY1 expression vector (YY1.V) or its corresponding empty vector (EV) harvested 24 and 48h after transfection. The study-based anti-YY1 antibody (ab109237) and two other antibodies (sc7341 and sc1703) were tested. GAPDH served as loading control.
